# Fluid balance and body composition analysis in female soccer players effect of the match

**DOI:** 10.1186/1550-2783-8-S1-P31

**Published:** 2011-11-07

**Authors:** Luca Gatteschi, F Angelini, A Bonuccelli, F Marzatico, M Rubenni, P Zeppilli

**Affiliations:** 1 Medical Section of Technical Sector, Italian football Federation (FIGC; 2 SINSEB

## Background

Dehydration refers to an imbalance in fluid dynamics when fluid intake doesn’t replenish water losses. Limiting dehydration during training and competition is a major concern for soccer players. A moderate exercise workout generally produces a 0.5to1.5 litre sweat loss over a 1 hour period, depending on training status and individual features. Changes in body weight (before/after match) indicate the extent of body loss during exercise, and the adequacy of fluid supply during the match. However, it’s also important to consider fluid shifts between different body compartments (intra vs. extracellular), and the influence of fluid loss and shifts on functional and subjective parameters and fatigue. The aim of the study was to assess individual sweat rates during a soccer match, and the relationships between sweat rates and both body composition change and rate of perceived exhaustion.

## Methods

Players of the Under 19 Italian National team, engaged in a friendly tournament (Spain, March 2009) took part in the study. The players were weighed naked immediately before and after the match, and the air temperature during the matches was respectively 14°, 19°, 19° C. The players were allowed to drink both water and a mineral-carbohydrate beverage (carbohydrate 4%), and we recorded the amount of fluid consumed by each player during warm up and match. Individual sweating rates were evaluated by dividing the decrease in body mass by the number of minutes played. Body composition was assessed by bioelectrical impedance analysis (BIA, Akern EFG device); data were collected in the morning, about 4 hours before the match and the day after. Rates of subjective fatigue were assessed by the Borg scale.

## Conclusion

Due to the small number of players engaged in the study, this has to be considered only the first step. However, it is possible to underline some salient findings: The evaluation of individual sweat rates was quite easy to perform but at the same time affordable and repeatable. The individual sweat rate (litres/hour) we recorded in some players were quite high. So it’s possible to suppose that those players may have difficulty maintaining an optimal fluid balance during the game. Identifying these players is important because they will need special drinking strategies in order to avoid dehydration and impaired thermoregulation. Body impedance analysis (BIA) showed: a) a shift of fluids, with a greater decrease in the extracellular compartment; b) a good correlation, although with a small number of subjects, between lower phase angle values (players with low physical condition and/or a late decrease of Body Cell Mass) and higher levels of subjective fatigue. Therefore, the BIA helps confirm our previous hypothesis about the possible role in monitoring physical conditions, with the capability to identify individuals who are at an increase risk of dehydration.

